# Integrating clinical and biochemical markers: a novel nomogram for predicting lacunes in cerebral small vessel disease

**DOI:** 10.3389/fnagi.2024.1404836

**Published:** 2024-08-23

**Authors:** Ning Li, Ya-Dong Hu, Ye Jiang, Li Ling, Chu-Han Wang, Jia-Min Shao, Si-Bo Li, Wei-Ying Di

**Affiliations:** Department of Neurology, Affiliated Hospital of Hebei University, Baoding, Hebei, China

**Keywords:** cerebral small vessel disease (CSVD), lacunes, nomogram, predictive model, clinical and biochemical markers, ROC curve analysis

## Abstract

**Background:**

Lacunes, a characteristic feature of cerebral small vessel disease (CSVD), are critical public health concerns, especially in the aging population. Traditional neuroimaging techniques often fall short in early lacune detection, prompting the need for more precise predictive models.

**Methods:**

In this retrospective study, 587 patients from the Neurology Department of the Affiliated Hospital of Hebei University who underwent cranial MRI were assessed. A nomogram for predicting lacune incidence was developed using LASSO regression and binary logistic regression analysis for variable selection. The nomogram’s performance was quantitatively assessed using AUC-ROC, calibration plots, and decision curve analysis (DCA) in both training (*n* = 412) and testing (*n* = 175) cohorts.

**Results:**

Independent predictors identified included age, gender, history of stroke, carotid atherosclerosis, hypertension, creatinine, and homocysteine levels. The nomogram showed an AUC-ROC of 0.814 (95% CI: 0.791–0.870) for the training set and 0.805 (95% CI: 0.782–0.843) for the testing set. Calibration and DCA corroborated the model’s clinical value.

**Conclusion:**

This study introduces a clinically useful nomogram, derived from binary logistic regression, that significantly enhances the prediction of lacunes in patients undergoing brain MRI for various indications, potentially advancing early diagnosis and intervention. While promising, its retrospective design and single-center context are limitations that warrant further research, including multi-center validation.

## Introduction

Cerebral small vessel disease (CSVD) represents a significant neurological condition that poses a substantial public health challenge globally, particularly prevalent among the elderly population (Wardlaw et al., 2019). It is closely associated with a spectrum of severe disease outcomes, including stroke and cognitive decline (Vermeer et al., 2003; Debette et al., 2019). Among various radiological manifestations of CSVD, lacunes hold particular clinical significance, typically resulting from occlusive ischemic injuries to small vessels (Wardlaw et al., 2013; Duering et al., 2023). These lacunes are not only markers of underlying vascular pathology but also predictors of future cerebrovascular events and cognitive impairments.

In the realm of neuroimaging, Magnetic Resonance Imaging (MRI) plays a pivotal role in identifying lacunes (Wardlaw et al., 2013; Duering et al., 2023). However, the detection capability of MRI is limited to the presence of established structural brain damage, implying that lacunes might not be accurately identified in the early stages of the lesion or when significant structural damage has not yet formed. Consequently, reliance solely on radiological findings could lead to missed or delayed detection, especially since lacunes are often asymptomatic in their early stages and can be easily overlooked (Mok and Kim, 2015). This underscores the need for a more comprehensive approach that integrates clinical history and laboratory findings to predict the presence of lacunes.

Our study addresses this challenge by developing a predictive model based on the analysis of a wide range of clinical and laboratory parameters from 587 individuals. The model aims to predict the presence of lacunes in cranial MRI, thereby facilitating early identification and intervention. Incorporating variables such as age, gender, history of hypertension, diabetes, stroke, along with blood test indicators, the model provides clinicians with a nuanced tool for risk assessment.

This predictive model represents a significant advancement in the early diagnosis of lacunes and offers a platform for understanding the complex interplay of various risk factors leading to the development of lacunes. The integration of clinical and laboratory data in predicting radiological outcomes signifies a major stride in personalized medicine and targeted interventions in the field of neurology.

Furthermore, the model’s ability to stratify patients based on their risk of developing lacunes has profound implications for preventive strategies against lacune. It enables healthcare providers to identify individuals at high risk and accordingly tailor management plans, potentially reducing the incidence of stroke and cognitive disorders associated with lacune.

## Method

### Study population and design

This retrospective study was conducted at the Neurology Department of the Affiliated Hospital of Hebei University, spanning from January 2020 to June 2022. We systematically collected and analyzed data from existing patient records, focusing on the period mentioned. The study’s retrospective nature allowed for the utilization of a large dataset, enabling a comprehensive analysis of pre-established data. Inclusion criteria were defined to ensure a representative sample from the patient population. These criteria included patients aged over 55 years and those with complete cranial magnetic resonance imaging (MRI) sequences, comprising T1-weighted axial, T2-weighted axial, FLAIR, and axial susceptibility-weighted images. Our study population was not limited to patients with specific diseases; any patient who had undergone a brain MRI for any reason, including those with normal MRI findings, was considered a potential participant. Conversely, exclusion criteria aimed to eliminate cases that could potentially confound the study outcomes, such as patients with poor-quality MRI images, significant stroke history, or severe comorbid conditions including but not limited to advanced cardiac, respiratory, renal, or hepatic diseases, and tumors. Cases suggestive of non-vascular origins of CSVD, like multiple sclerosis or central nervous system demyelinating diseases, were also excluded, alongside those with insufficient clinical or laboratory data. To ensure the confidentiality and privacy of patient data, all records were anonymized before analysis, with all personal identifiers removed. The data handling process was conducted in strict compliance with data protection regulations. The study protocol was rigorously reviewed and approved by the Institutional Review Board (IRB) of the Affiliated Hospital of Hebei University under Approval Number HDFYLL-KY-2023-060. This study was conducted in strict accordance with the ethical standards set forth in the 1964 Declaration of Helsinki and its later amendments. Ethical approval for this research involving human participants was obtained from the appropriate ethics committee. We hereby confirm that all national laws and regulations concerning ethical standards in research were observed, ensuring that the conduct of the research complied with both international and national ethical standards.

### MRI acquisition and assessment

Participants underwent brain MRI using a 1.5 T MRI scanner (Siemens, Munich, Germany). Standardized MRI sequences included axial T1-weighted, sagittal T2-weighted fluid-attenuated inversion recovery (FLAIR), and axial susceptibility-weighted images. The imaging parameters were as follows: slice thickness set at 5 mm with a 1-mm interslice gap; for T1-weighted spin echo, repetition time (TR)/echo time (TE) were 700/11 ms; for T2-weighted fast spin echo, TR/TE were 5200/120 ms; and for FLAIR, TR/TE and inversion time were 8500/127 ms and 2,300 ms, respectively. This protocol aimed to identify the radiological manifestations of lacunes. The evaluation was carried out according to the guidelines for reporting vascular changes in neuroimaging (STRIVE) (Wardlaw et al., 2013; Duering et al., 2023) Lacunes were identified as round or ovoid subcortical cavities, ranging from 3 to 15 mm in diameter, resembling cerebrospinal fluid (CSF) in signal characteristics. On fluid-attenuated inversion recovery (FLAIR) imaging, these vascular-origin lacunes typically exhibit a central CSF-like hypointense core surrounded by a hyperintense rim. The presence and location of lacunes were assessed by two experienced neurologists, Y. Jiang and L. Ling, who were blinded to the clinical data of the participants. This approach aimed to minimize assessment bias. To ensure reliability of the assessments, the interrater agreement was quantified using the intraclass correlation coefficient (ICC), with a substantial agreement indicated by an ICC of 0.85.

### Clinical blood biochemistry assessment

In the retrospective analysis of our patient cohort, we meticulously compiled a comprehensive dataset encompassing a broad spectrum of clinical parameters. This dataset included an extensive range of blood biochemistry markers, such as complete blood count, renal function indicators, electrolyte levels, coagulation profiles, both random and fasting blood glucose measurements, liver function tests, lipid profiles, cardiac enzyme panels, thyroid function assessments, and homocysteine levels. Altogether, the study incorporated 81 distinct laboratory parameters, covering vital aspects of patient health and providing a thorough clinical picture for each enrolled individual.

### Clinical evaluation

In our study, a thorough clinical evaluation was performed for each participant, which included the collection of demographic information such as age and sex, along with a comprehensive medical history. In the medical history assessment, particular emphasis was placed on key health conditions including hypertension, diabetes, and hypercholesterolemia, as well as on the history of carotid artery atherosclerosis and stroke. The diagnostic criteria for these conditions were rigorously adhered to as follows: Hypertension was defined as having a systolic blood pressure of 140 mmHg or higher, a diastolic blood pressure of 90 mmHg or higher, or being under current treatment with antihypertensive medications. Diabetes was established based on fasting blood glucose levels of 7.0 mmol/L or above, 2-h post-oral glucose tolerance test (OGTT2h) readings exceeding 11.1 mmol/L, or ongoing treatment with hypoglycemic agents. Hypercholesterolemia was identified if total cholesterol levels exceeded 5.2 mmol/L (200 mg/dL) or low-density lipoprotein (LDL) cholesterol levels surpassed 3.4 mmol/L (130 mg/dL). Carotid atherosclerosis is defined as a history of carotid atherosclerosis or evidence of increased intima-media thickness or the presence of plaques in the carotid arteries, as indicated by carotid ultrasound examination performed during hospitalization.

### Statistical analysis

A cohort comprising 587 patients underwent random allocation into two distinct sets: the training dataset, consisting of 412 individuals, and the validation dataset, consisting of 175 individuals. This allocation adhered to a predetermined ratio of 7:3. In the development of the model, the conversion of continuous variables into categorical ones was adopted, a strategy commonly employed in the literature for risk prediction models to facilitate ease of interpretation and to bolster generalizability. This approach is widely recognized for its clinical applicability and broad acceptance (Bennette and Vickers, 2012; Barrio et al., 2017). The determination of cutoff values for these continuous variables was guided by using R software, In cases where explicit cutoffs were not readily available, parameters were systematically categorized into binary or tertiary groups. Categorical variables were presented as frequencies with corresponding percentages (%). To compare the baseline characteristics between lacune group and non-lacune group, we employed statistical tests appropriate for the data types. Specifically, we used the χ2 test or Fisher’s exact test for categorical variables. For variable selection in the training dataset, we employed the Least Absolute Shrinkage and Selection Operator (LASSO) regression, guided by cross-validation. To strike a balance between model complexity and predictive accuracy, the regularization parameter (lambda) was chosen based on the lambda.1se criterion, which corresponds to the value within one standard error of the minimum mean cross-validated error. This conservative approach favors a simpler model with fewer predictors, thereby reducing the risk of overfitting while maintaining sufficient predictive power. In our analysis, variables that showed significant associations in the LASSO regression (with non-zero coefficients) were selected for further exploration. These variables were then integrated into a binary logistic regression to pinpoint independent predictors of lacunes. Based on the identified predictors, we developed a nomogram to facilitate the visual interpretation of risk factors. The performance of the nomogram was evaluated through the Receiver Operating Characteristic Curve (ROC) analysis, specifically examining the Area Under the Curve (AUC) to gauge its predictive strength. Additionally, calibration plots were generated to juxtapose the predicted outcomes against actual probabilities, offering insights into the model’s accuracy. The clinical usefulness of the predictive model was further assessed using Decision Curve Analysis (DCA), which helped in evaluating the net benefits. All statistical procedures were executed using R software (version 4.3.0). For inferential statistics, a threshold of *p* < 0.05 (two-tailed) was set to determine statistical significance.

## Results

### Baseline characteristics

**Figure 1:**
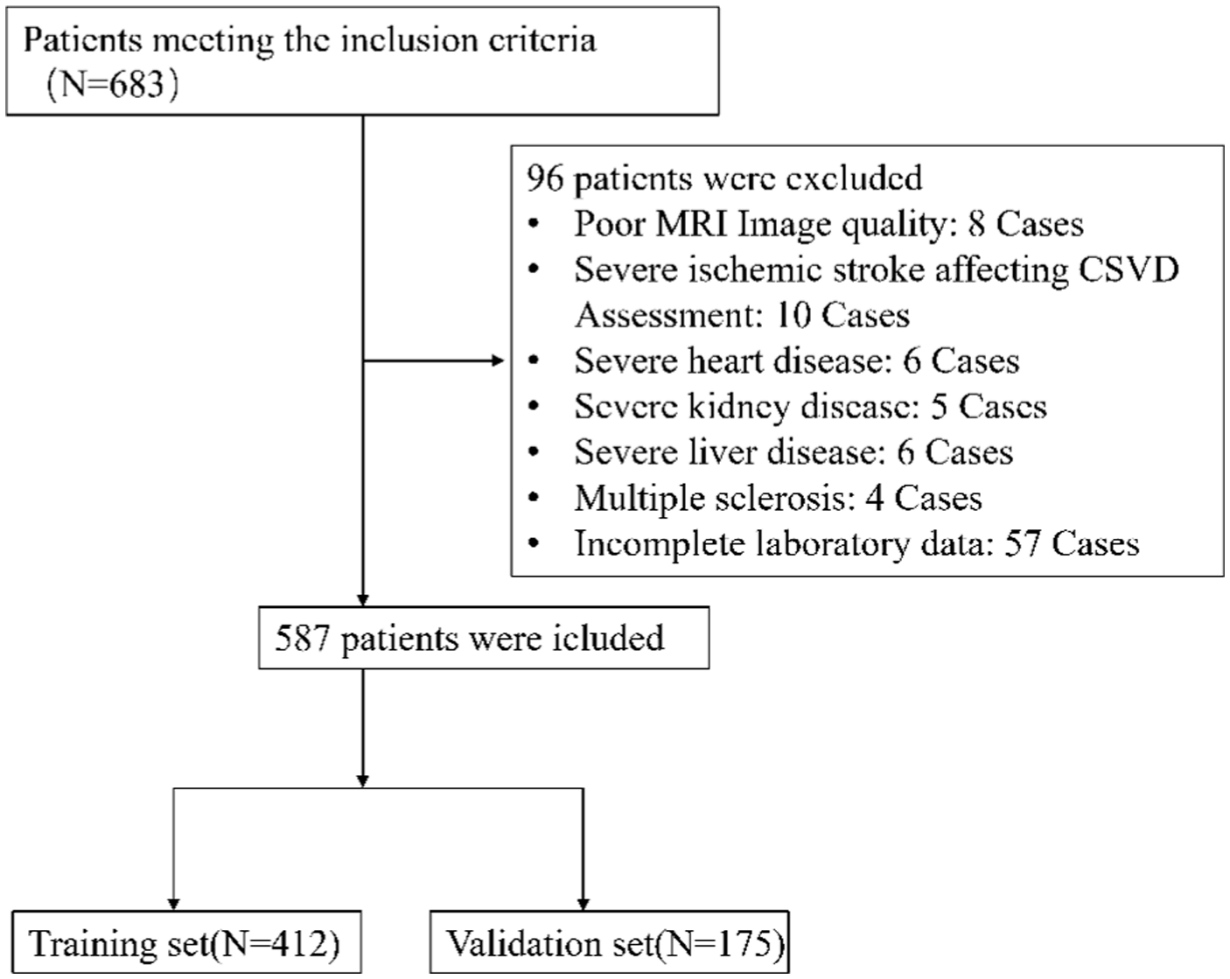
Flow diagram of the selection of eligible patients.

From January 2020 to June 2022, our study initially enrolled 683 patients who satisfied the inclusion criteria. However, upon further evaluation, 96 patients were identified as meeting the exclusion criteria and were thus removed from the study. This led to a final cohort of 587 patients who were deemed eligible for data analysis, as depicted in Figure 1. The study was structured with a training set comprising 412 individuals, while the remaining 175 formed the validation set. Table 1 presents the baseline characteristics of the patients, distinguishing between those with lacunar and non-lacunar conditions.

**Table 1:** Baseline characteristics: comparing lacune patients with non-lacune patients.

Variables	Total (*n* = 587)	Non-Lacune (*n* = 403)	Lacune (*n* = 184)	*p*
Gender, *n* (%)				< 0.001
Female	307 (52)	242 (60)	65 (35)	
Male	280 (48)	161 (40)	119 (65)	
Age (years), *n* (%)				< 0.001
<=59	204 (35)	148 (37)	56 (30)	
60–67	202 (34)	156 (39)	46 (25)	
> = 68	181 (31)	99 (25)	82 (45)	
History of stroke, *n* (%)				< 0.001
No	443 (75)	334 (83)	109 (59)	
Yes	144 (25)	69 (17)	75 (41)	
Carotid atherosclerosis, *n* (%)				< 0.001
No	245 (42)	192 (48)	53 (29)	
Yes	342 (58)	211 (52)	131 (71)	
Hypertension, *n* (%)				< 0.001
Normal	189 (32)	159 (39)	30 (16)	
Level1	109 (19)	83 (21)	26 (14)	
Level2	251 (43)	141 (35)	110 (60)	
Level3	38 (6)	20 (5)	18 (10)	
Diabetes, *n* (%)				0.033
No	437 (74)	311 (77)	126 (68)	
Yes	150 (26)	92 (23)	58 (32)	
Homocysteine (μmol/l), *n* (%)				< 0.001
<=26	513 (87)	371 (92)	142 (77)	
>26	74 (13)	32 (8)	42 (23)	
White blood cell count (×109), *n* (%)				0.015
<=6.73	294 (50)	216 (54)	78 (42)	
>6.73	293 (50)	187 (46)	106 (58)	
Monocyte count (×109), *n* (%)				0.008
<=0.44	295 (50)	218 (54)	77 (42)	
>0.44	292 (50)	185 (46)	107 (58)	
Basophil Count (×109), *n* (%)				0.014
<=0.03	394 (67)	284 (70)	110 (60)	
>0.03	193 (33)	119 (30)	74 (40)	
Urea (mmol/L), *n* (%)				< 0.001
<=4.8	203 (35)	163 (40)	40 (22)	
>4.8	384 (65)	240 (60)	144 (78)	
Uric Acid (μmol/L), *n* (%)				0.022
<=298	298 (51)	218 (54)	80 (43)	
>298	289 (49)	185 (46)	104 (57)	
Creatinine (μmol/l), *n* (%)				< 0.001
<=78	479 (82)	356 (88)	123 (67)	
>78	108 (18)	47 (12)	61 (33)	
Carbon dioxide content in plasma (mmol/L), *n* (%)				0.008
<=27	384 (65)	249 (62)	135 (73)	
>27	203 (35)	154 (38)	49 (27)	
Fibrinogen (g/L), *n* (%)				0.029
<=2.88	296 (50)	216 (54)	80 (43)	
>2.88	291 (50)	187 (46)	104 (57)	
Globulin (g/L), *n* (%)				0.001
<=26	340 (58)	252 (63)	88 (48)	
>26	247 (42)	151 (37)	96 (52)	
Albumin to globulin ratio, *n* (%)				< 0.001
<=1.53	336 (57)	206 (51)	130 (71)	
>1.53	251 (43)	197 (49)	54 (29)	
Free thyroxine (ng/ml), *n* (%)				0.023
<=1.22	304 (52)	222 (55)	82 (45)	
>1.22	283 (48)	181 (45)	102 (55)	
Total cholesterol (mmol/L), *n* (%)				0.013
<=4.5	295 (50)	188 (47)	107 (58)	
>4.5	292 (50)	215 (53)	77 (42)	
High-density lipoprotein, *n* (%)				< 0.001
<=1	180 (31)	100 (25)	80 (43)	
>1	407 (69)	303 (75)	104 (57)	
Low-density lipoprotein (mmol/L), *n* (%)				0.007
<=2.89	295 (50)	187 (46)	108 (59)	
>2.89	292 (50)	216 (54)	76 (41)	
Lipoprotein (a) (mg/L), *n* (%)				0.004
<=185	295 (50)	219 (54)	76 (41)	
>185	292 (50)	184 (46)	108 (59)	
Apolipoprotein A1(g/L), n (%)				< 0.001
<=1.03	296 (50)	183 (45)	113 (61)	
>1.03	291 (50)	220 (55)	71 (39)	
Apolipoprotein E (mg/L), *n* (%)				0.002
<=37	296 (50)	185 (46)	111 (60)	
>37	291 (50)	218 (54)	73 (40)	
Monocyte-to-HDL ratio, *n* (%)				< 0.001
<=0.27	127 (22)	108 (27)	19 (10)	
>0.27	460 (78)	295 (73)	165 (90)	
Neutrophil-to-HDL ratio, *n* (%)				0.002
<=3.97	293 (50)	219 (54)	74 (40)	
>3.97	294 (50)	184 (46)	110 (60)	
Lymphocyte-to-monocyte ratio, *n* (%)				0.002
<=3.47	294 (50)	184 (46)	110 (60)	
>3.47	293 (50)	219 (54)	74 (40)	

In our study, we compared two groups: the lacune group (*n* = 184)and the non-lacune group (*n* = 403). We found significant differences (*p* < 0.05) in 27 out of the 88 analyzed variable between these two groups, while the remaining 61 biomarkers showed no significant differences. The 27 biomarkers with significant differences included: Gender, Age, History of Stroke, Carotid atherosclerosis, Hypertension, Diabetes, Homocysteine, White Blood Cell Count, Monocyte count, Basophil Count, Urea, Uric Acid, Creatinine, Carbon Dioxide Content in Plasma, Fibrinogen, Globulin, Albumin to Globulin Ratio, Free Thyroxine, Total Cholesterol, High-Density Lipoprotein, Low-Density Lipoprotein, Lipoprotein (a), Apolipoprotein A1, Apolipoprotein E, Monocyte-to-HDL Ratio, Neutrophil-to-HDL Ratio, Lymphocyte-to-Monocyte Ratio (Table 1). The other 61 biomarkers showed no significant differences between the two groups including: Hyperlipidemia, Red Blood Cell Count, Hemoglobin, Hematocrit, Mean Corpuscular Volume, Mean Corpuscular Hemoglobin, Mean Corpuscular Hemoglobin Concentration, Platelet Count, Platelet Distribution Width, Mean Platelet Volume, Plateletcrit, Neutrophil Percentage, Lymphocyte Percentage, Monocyte Percentage, Eosinophil Percentage, Basophil Percentage, Neutrophil Count, Lymphocyte Count, Eosinophil Count, Platelet Large Cell Ratio, Random Glucose, Potassium, Sodium, Chloride, Calcium, Magnesium, Serum Phosphate, Prothrombin Time, Prothrombin Time Ratio, International Normalized Ratio, Activated Partial Thromboplastin Time, Thrombin Time, Fasting Glucose, Hemoglobin A1c, Triglycerides, Very Low-Density Lipoprotein, Apolipoprotein B100, ApoA1 vs. ApoB100 Ratio, Creatine Kinase, Creatine Kinase-MB, Alpha-Hydroxybutyrate Dehydrogenase, Alanine Aminotransferase, Aspartate Aminotransferase, Lactate Dehydrogenase, Alkaline Phosphatase, Gamma-Glutamyl Transferase, Total Protein, Albumin, Total Bilirubin, Direct Bilirubin, Unconjugated Bilirubin, Bile Acid, Triiodothyronine, Thyroxine, Free Triiodothyronine, Thyroid-Stimulating Hormone, Systemic Immune-Inflammatory Index, Systemic Inflammation Response Index, Neutrophil-to-Lymphocyte Ratio, Neutrophil-to-Monocyte Ratio, Lymphocyte-to-HDL Ratio.

### Variable selection

**Figure 2:**
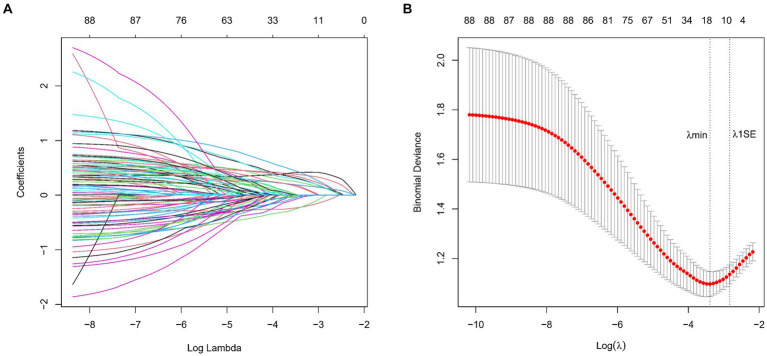
**(A)** LASSO Coefficient Profiles for lacune Clinical Predictors. This figure presents the coefficient profiles of 88 features considered in the LASSO model for predicting the risk of lacune. The graph displays how each feature’s coefficient varies with the log of lambda (log(lambda)), demonstrating the shrinkage effect of the LASSO technique. **(B)** LASSO Regression Cross-Validation Results. This figure illustrates the evaluation of model performance under various regularization parameters λ through cross-validation in LASSO regression. A vertical dashed line on the left side represents λmin, which corresponds to the model with the best performance. On the right side, another vertical dashed line denotes λ1SE, representing a slightly sparser model. The numbers of selected variables are annotated above each line.

We collected data covering a total of 88 variables, including age, gender, medical history, and laboratory tests. In our study, the LASSO regression was implemented using the “glmnet package” in R with a 10-fold cross-validation approach to optimize the regularization parameter λ. We selected the value of λ based on the 1SE (one standard error) criterion, which aims at choosing a simpler model with a performance within one standard error of the minimum cross-validation error. This approach helped in striking a balance between model complexity and prediction accuracy, enabling us to identify the most relevant predictors for our model while controlling for overfitting (Figures 2A,B). Through LASSO regression, we selected 9 indicators: Gender, Age, History of Stroke, Carotid Atherosclerosis, Hypertension, Creatinine, High-Density Lipoprotein, Homocysteine, Monocyte-to-HDL Ratio (Table 2). The variables with non-zero coefficients in the LASSO regression model were considered to be related to lacune.

**Table 2:** Coefficients and lambda.1SE value of the LASSO regression.

Variable	Coefficients	Lambda.1SE
Gender	0.0225	0.0776
History of stroke	0.0647	
Carotid atherosclerosis	0.022	
Hypertension	0.0245	
Creatinine	0.0892	
High-density lipoprotein	−0.023	
Homocysteine	0.0433	
Monocyte-to-HDL ratio	0.0223	
Age	0.0349	

This table concisely encapsulates the LASSO regression analysis outcomes, spotlighting pivotal variables like Gender, Stroke, Carotid Atherosclerosis, Hypertension, Creatinine, High-Density Lipoprotein, Homocysteine, Monocyte-to-HDL Ratio, and Age. It includes the critical lambda.1SE value for model selection, indicative of the model’s reliability and predictive precision. This analysis is integral to our predictive model’s development, underscoring the significance of these variables in evaluating the risk of lacunes.

### Multivariable analyses

In the binary logistic regression analysis, we included the 9 variables identified from the LASSO regression. The analysis revealed that seven of these variables—Gender, Age, History of Stroke, Carotid Atherosclerosis, Hypertension, Creatinine, and Homocysteine—were significantly associated with the risk of lacune (*p* < 0.05), as detailed in Table 3. These results indicate that these seven variables are independent clinical predictors of lacune. The remaining two variables, High-Density Lipoprotein (HDL) and Monocyte-to-HDL Ratio, were included in the model but did not show a statistically significant association in this analysis.

**Table 3:** Binary logistic regression analysis.

	*B*	SE	OR	CI	*Z*	*P*
Gender	0.624	0.251	1.87	1.14–3.05	2.489	0.013
History of stroke	0.638	0.263	1.89	1.13–3.17	2.426	0.015
Carotid atherosclerosis	0.576	0.256	1.78	1.08–2.94	2.248	0.025
Hypertension	0.562	0.139	1.75	1.34–2.30	4.039	0.000
Creatinine	0.746	0.302	2.11	1.17–3.81	2.473	0.013
Homocysteine	0.637	0.359	1.89	1.14–3.82	1.777	0.036
Age	0.476	0.15	1.61	1.20–2.16	3.181	0.001

The results of the binary logistic regression analysis, which was conducted on 9 variables initially identified through LASSO regression as potential predictors for lacune. Out of these, 7 variables were retained in the final lacune diagnostic model, namely (Gender, History of Stroke, carotid atherosclerosis, Hypertension, Creatinine, Homocysteine, Age). The remaining 2 variables that were not included in the final model due to p > 0.05 are (High-Density Lipoprotein, Monocyte-to-HDL Ratio). The table details the regression coefficient (B), standard error (SE), odds ratio (OR), 95% confidence interval (95% CI), Z-value, and p-value for each of the 7 included factors.

### Predictive model development

**Figure 3:**
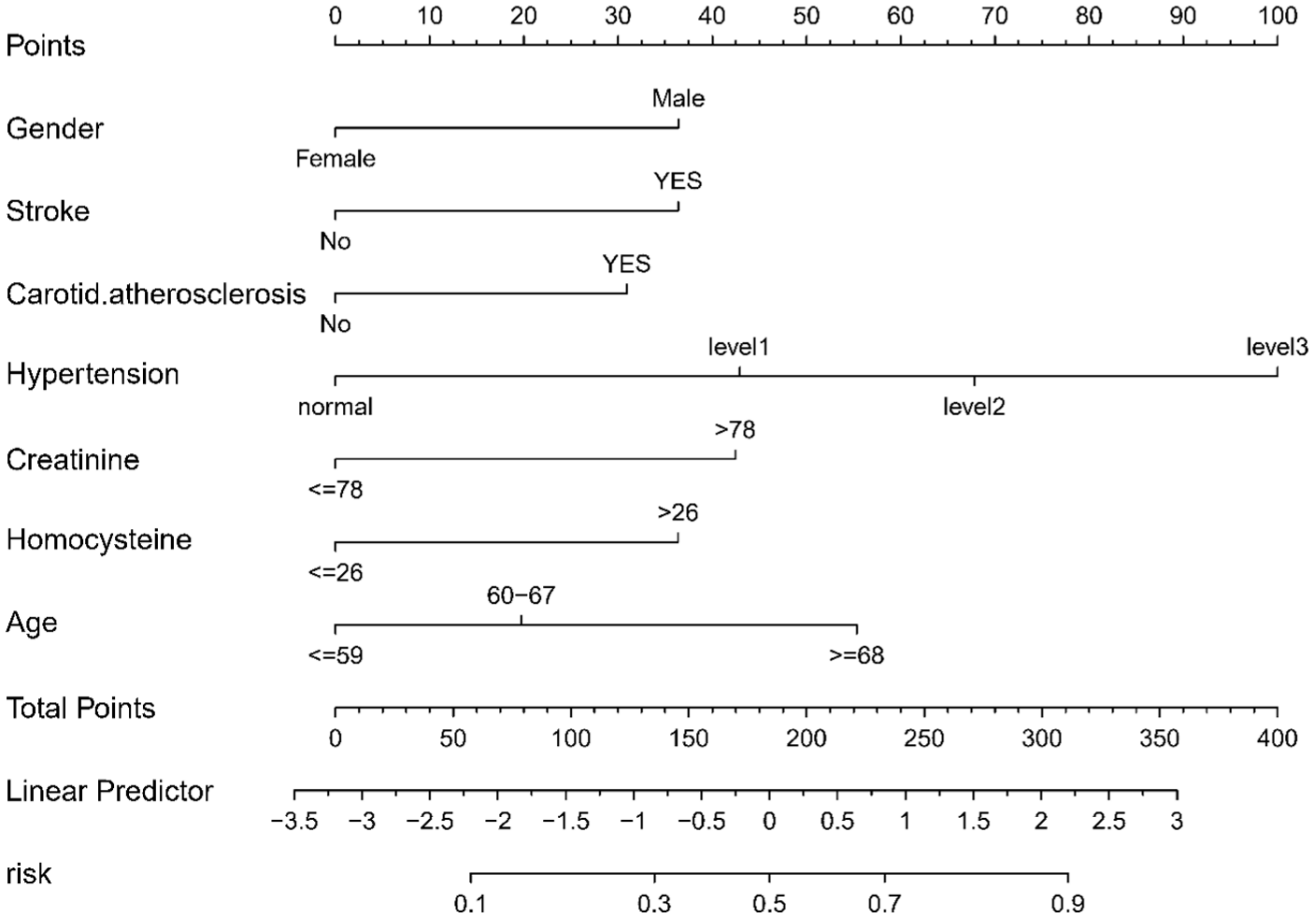
Nomogram for Predicting the Risk of lacune. For all patients, points are calculated based on seven indicators by aligning them with corresponding point scales. The cumulative sum of points is then located on the “Total Points” axis. Subsequently, the risk of lacune, as determined by the nomogram, corresponds to the probability indicated on the “lacune” scale corresponding to the “Total Points”.

In this study, binary logistic regression analysis was employed to identify key variables associated with the risk of lacunes. Seven variables were selected based on their statistical significance: Gender, Age, History of Stroke, Carotid Atherosclerosis, Hypertension, Creatinine, and Homocysteine. These variables were then used to construct a nomogram, as depicted in Figure 3. The nomogram operates by assigning point values to each variable based on their calculated beta coefficients, reflecting their proportional prognostic impact. The total points accrued from each variable are then used to estimate the patient’s probability of developing lacunes. This probability is derived from the total point’s projection on the probability scale provided at the bottom of the nomogram. The inclusion of diverse variables, ranging from biochemical markers like Creatinine and Homocysteine to clinical features such as the presence of Carotid Atherosclerosis, allows for a comprehensive assessment of lacunes risk. This approach enables healthcare providers to make more informed decisions regarding patient care and risk management.

### Nomogram validation

**Figure 4:**
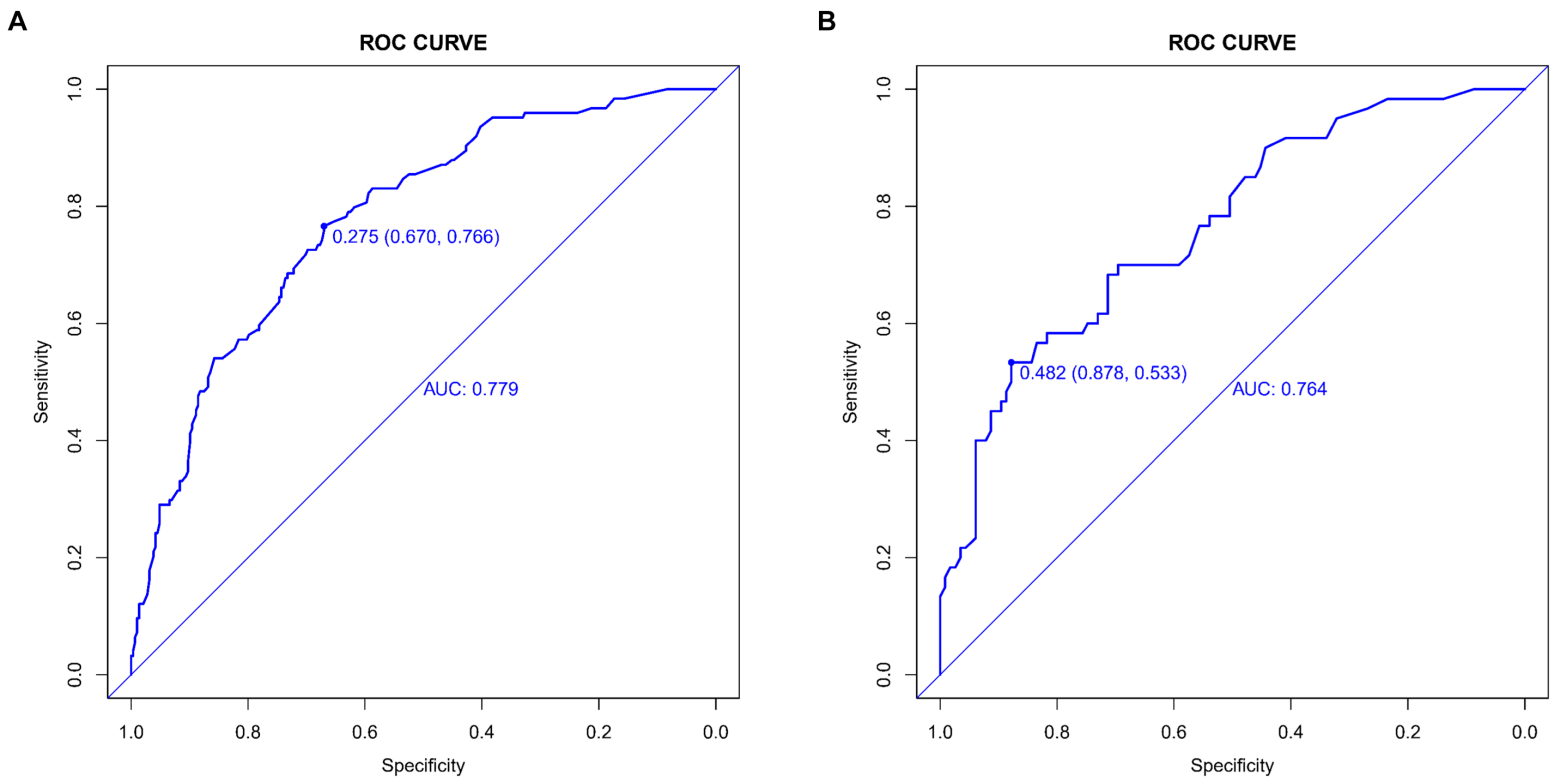
Receiver operating characteristic (ROC) curves for lacune Predictive Model in Training Set **(A)** and Validation Set **(B)**. The ROC curves plot the sensitivity against the specificity for various threshold levels.

**Figure 5:**
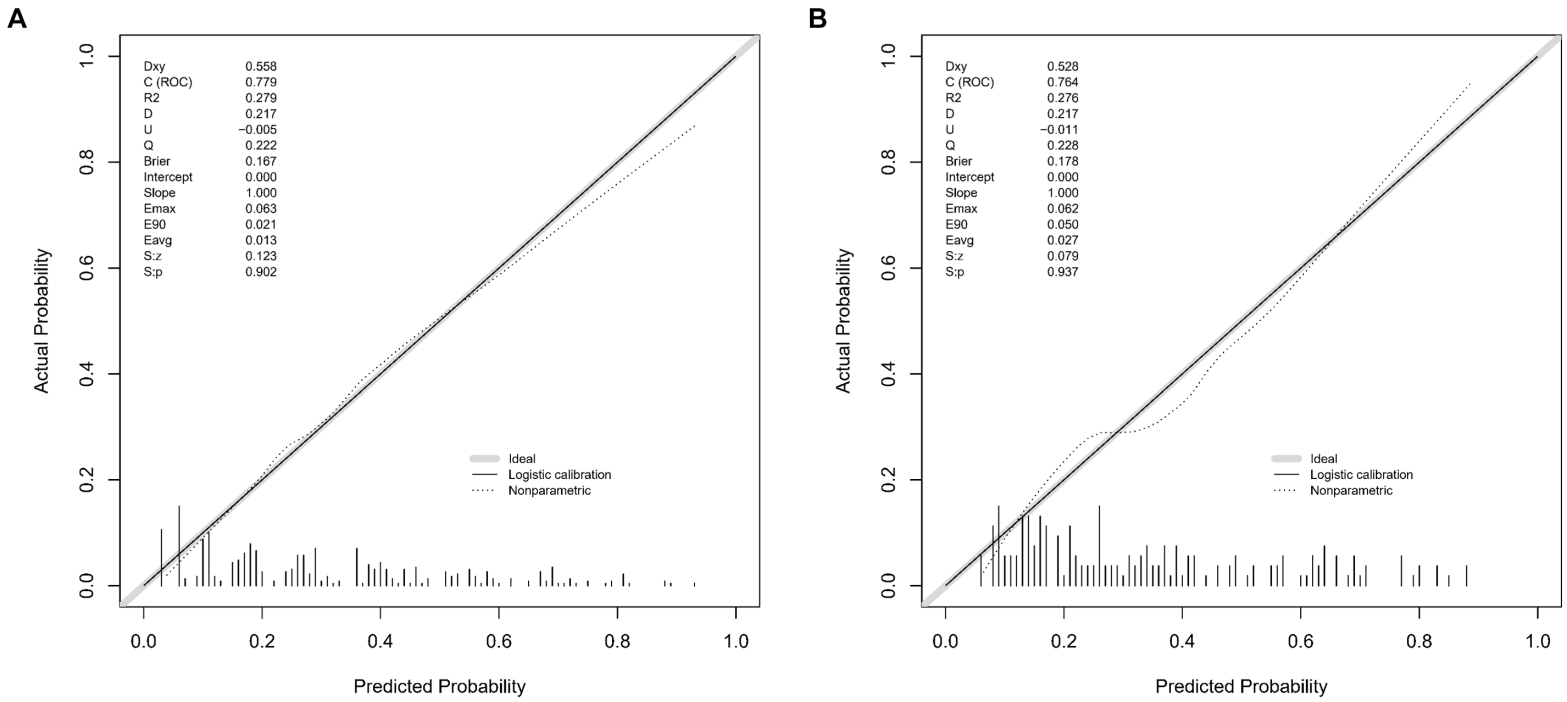
Calibration plots for the lacune predictive model in the training set **(A)** and validation set **(B)**. The x-axis represents the predicted probability of lacune occurrence, while the y-axis represents the observed frequency of lacunes. The diagonal 45-degree line indicates perfect calibration, where predicted probabilities exactly match the observed outcomes. The dashed line represents the performance of our nomogram, with deviations from the diagonal line illustrating the degree of miscalibration. These plots demonstrate the accuracy and reliability of our predictive model in both the training and validation cohorts, with the calibration curve closer to the diagonal indicating better predictive accuracy.

**Figure 6:**
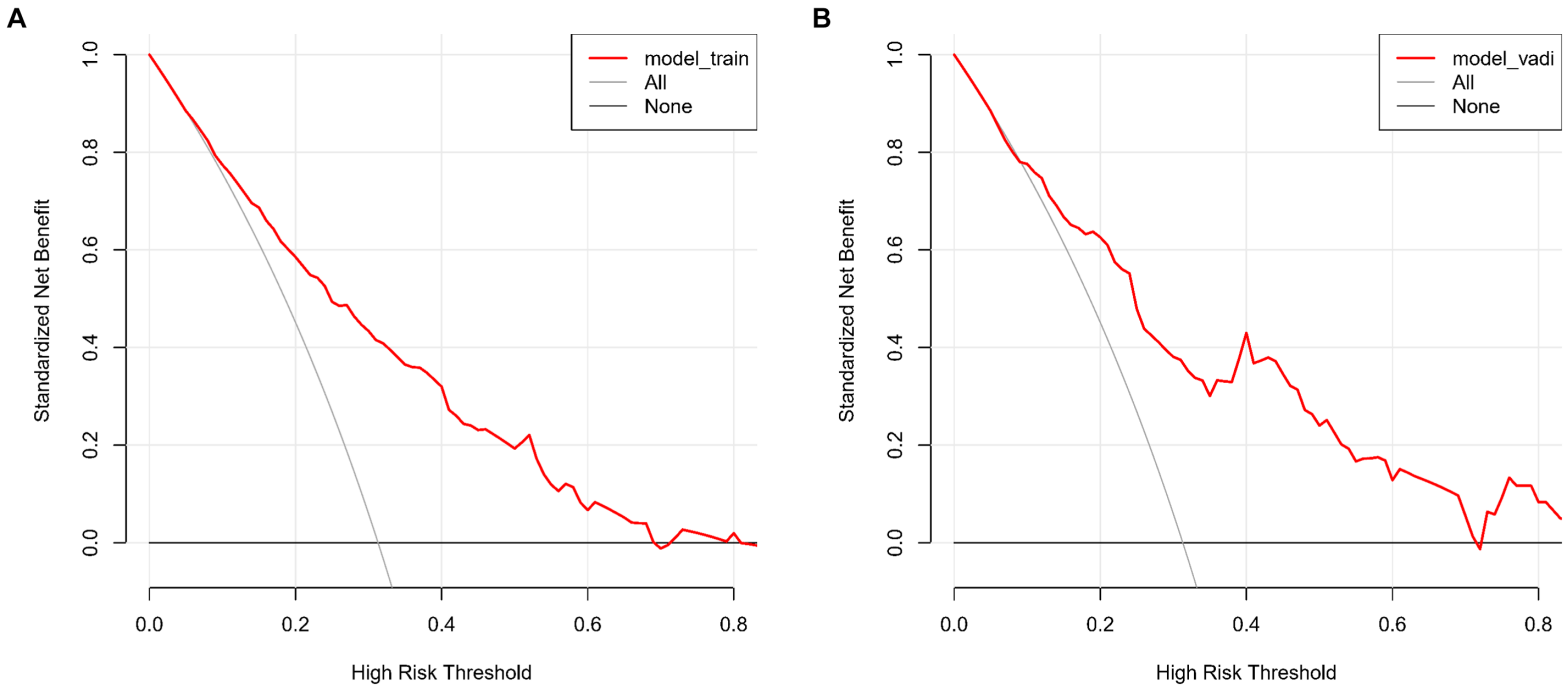
**(A,B)** show the Decision Curve Analysis (DCA) for our lacune predictive model on both the training **(A)** and validation sets **(B)**. The graphs illustrate the net benefit across various threshold probabilities of using our model versus either treating all or no patients. The model’s curves demonstrate its clinical value by offering a balanced approach to making treatment decisions, deviating from the extremes represented by the horizontal (no treatment) and oblique (universal treatment) lines.

The lacunar infarction risk prediction nomogram underwent thorough validation using Receiver Operating Characteristic (ROC) curves, calibration plots, and Decision Curve Analysis (DCA) across both the training and validation cohorts. In the ROC curve analysis, the training set demonstrated an AUC of 0.779 (95% CI: 0.731–0.826), and the validation set an AUC of 0.764 (95% CI: 0.691–0.838), indicating the nomogram’s consistent ability to differentiate risk levels of developing lacunar infarctions. Specifically, in the training set, the sensitivity was 67.0%, and the specificity was 76.6%. In the validation set, the sensitivity further improved to87.8%, while the specificity was53.3% (Figures 4A,B). Calibration analysis revealed an alignment between the predicted and observed outcomes in both cohorts, with intercepts and slopes at 0.000 and 1.000, respectively (Figures 5A,B). The Brier scores were 0.167 for the training set and 0.178 for the validation set, reflecting the model’s accuracy in each dataset. The Decision Curve Analysis (Figures 6A,B) showed the model’s curve notably deviating from the extremes, suggesting its clinical benefit compared to baseline strategies. These results collectively demonstrate the nomogram’s accuracy and reliability in predicting lacunar infarction risk, affirming its potential utility in clinical settings.

## Discussion

This study successfully established a clinical and laboratory parameter-based model for predicting lacune, demonstrating robust predictive capabilities across two independent cohorts. By integrating variables such as gender, age, history of stroke, carotid atherosclerosis, hypertension, creatinine, and homocysteine, our model provides a comprehensive risk assessment for the occurrence of lacune. We emphasize the importance of a comprehensive assessment in predicting CSVD, extending beyond the traditionally focused radiological features to include clinical and biochemical markers. Our model underscores the correlation between these parameters and lacune development, offering new insights into the pathophysiology of CSVD. The identification of age and hypertension as predictors corroborates findings by scholars like Cannistraro et al. (2019) and Joutel (2020), who noted hypertension’s significant role in CSVD pathogenesis. Similarly, Pantoni (2010) and Wardlaw et al. (2013) have highlighted the influence of age in CSVD, reinforcing the relevance of these factors in lacune prediction and clinical management.

Elevated creatinine levels were identified as predictors of lacune, aligning with studies by Akoudad et al. (2015) and Xiao et al. (2015), which observed associations between renal impairment and CSVD or lacunar stroke. This suggests the indirect influence of renal function on lacune risk via cerebral vasculature, highlighting the importance of monitoring creatinine levels in lacune risk assessment. Our model also recognizes male gender as a significant predictor, resonating with Jiménez-Sánchez et al. (2021)'s findings on sex differences in CSVD. This suggests a possibly higher risk or distinct pathophysiological responses in males leading to lacune development, indicating the need for gender-specific approaches in lacune management and further investigation into underlying mechanisms. Furthermore, the role of homocysteine as a predictive factor for lacune aligns with the research by Nam et al. (2019), Rutten-Jacobs et al. (2016), and Kloppenborg et al. (2014), reinforcing its potential as a biomarker and its interaction with genetic factors in CSVD and lacune formation. Lastly, our study highlights prior stroke history and carotid atherosclerosis as key predictors for lacunar infarctions, suggesting a link between previous cerebrovascular events and the development of small vessel disease. This highlights the need for comprehensive management of carotid artery disease and vigilant monitoring in patients with a history of stroke, providing novel strategies for lacune prevention.

Our model represents a significant advance in the field, particularly due to the scarcity of similar studies. It enhances predictive accuracy and raises the likelihood of early detection of lacune through integrated analysis, crucial for early intervention and prevention of cerebral small vessel disease progression. However, our model has limitations. The retrospective nature of the study may introduce selection bias. Additionally, being a single-center study might affect the model’s generalizability. Furthermore, the impact of certain variables, such as lifestyle factors and genetic background, may not have been fully captured. Additionally, our nomogram has not yet been validated using other comparable external data sources. This limitation should be addressed in future research by validating the model with multicenter data to ensure its broader applicability. Future research should consider expanding the sample size and incorporating multi-center data to validate the model’s wider applicability. Exploring additional potential predictors, including lifestyle and genetic factors, and the model’s applicability across different ethnic and age groups, is also imperative. Our model can serve as a powerful tool for clinicians in assessing patients’ risk of lacune, especially for those whose radiological findings are inconclusive but clinical and biochemical markers indicate high risk. It could aid in more precise risk assessment and early intervention.

## Conclusion

In summary, our predictive model for lacunes, integrating multiple clinical and laboratory parameters, demonstrates notable predictive capabilities. However, it is important to note that the model’s applicability is specific to predicting lacunes in patients undergoing brain MRI for various indications, and not broader manifestations of CSVD. Despite its limitations, such as the retrospective nature and single-center data source, and the lack of validation using external data sources, the model offers a novel perspective in predicting lacunes, contributing valuable insights for future research and clinical practice. Future endeavors should focus on expanding sample sizes, incorporating multi-center studies, and exploring additional influencing factors, to further validate and enhance the model’s utility. This work lays the groundwork for advancing personalized treatment strategies in neurology, particularly for patients at high risk of lacunes whose radiological findings may be inconclusive but clinical and biochemical markers indicate elevated risk.

## Data Availability

The raw data supporting the conclusions of this article will be made available by the authors, without undue reservation.
